# Differences and overlaps between Phd studies in diagnostic microbiology in industrial and academic settings

**DOI:** 10.1007/s00430-019-00643-4

**Published:** 2019-11-29

**Authors:** Alex van Belkum, Andreu Coello Pelegrin, Rucha Datar, Manisha Goyal, Mattia Palmieri, Caroline Mirande, Valérie Chalansonnet, Sylvain Orenga

**Affiliations:** 1 Data Analytics Department, Biomérieux, 3 Route de Port Michaud, 38390 La Balme Les Grottes, France; 2 Clinical Unit, Biomérieux, 3 Route de Port Michaud, 38390 La Balme Les Grottes, France

**Keywords:** Infectious diseases, Diagnostics, PhD studies, Industry, Academia

## Abstract

Industrial and academic needs for innovation and fundamental research are essential and not widely different. Depending on the industrial setting, research and development (R&D) activities may be more focused on the developmental aspects given the need to ultimately sell useful products. However, one of the biggest differences between academic and industrial R&D will usually be the funding model applied and the priority setting between innovative research and product development. Generalizing, companies usually opt for development using customer- and consumer-derived funds whereas university research is driven by open innovation, mostly funded by taxpayer’s money. Obviously, both approaches require scientific rigor and quality, dedication and perseverance and obtaining a PhD degree can be achieved in both settings. The formal differences between the two settings need to be realized and students should make an educated choice prior to the start of PhD-level research activities. Intrinsic differences in scientific approaches between the two categories of employers are not often discussed in great detail. We will here document our experience in this field and provide insights into the need for purely fundamental research, industrial R&D and current mixed models at the level of European funding of research. The field of diagnostics in clinical bacteriology and infectious diseases will serve as a source of reference.

## The infectious disease diagnostic research arena

Clinical and laboratory-based diagnostics for bacterial infectious diseases are urgent requirements for better management of patients suffering from infections [1]. Four main innovation and research and development (R&I and R&D) fields can be identified. First and foremost, there is fundamental research on disease invoking pathogens and their pathogenic mechanisms employed. Such research helps identify new, emerging pathogens and is key in the identification of novel diagnostic markers [2]. Second, there is the taxonomic and technical research into the actual detection and identification of pathogens. Taxonomy is required to substantiate the novelty of emerging pathogens and to make sure there are clear markers for distinguishing all relevant human pathogens. The technologies currently used to identify microbial species are mostly growth-based, biochemical, often in combination with metabolic and phenotypic analyses, proteomic (e.g. mass spectrometry-based) or molecular using methods which are based on species-differentiating gene or genome sequences [3]. The third research domain for bacterial microbes is focused on defining their susceptibility and resistance towards the most frequently used therapeutic antibiotics. Again, technologies to detect and characterize the many resistance mechanisms that have already been defined can be phenotypic, genotypic or a combination of the two [4]. Fourth and final, there is epidemiological research. This is geared towards the elucidation of local and international spreading patterns for microorganisms and should help track-and-trace sources of infection in a reliable manner to prevent or stop outbreaks and interrupt environmental persistence [5].

New diagnostic approaches have significantly changed the field of infectious diseases over the past decades. Each of the recent revolutions, without exception, resulted from successes in one of the four priority fields listed above (see Box 1 for some more detail). Much of the progress was due to the transformational efforts and the high quality of work performed by many PhD students. Obviously, when aiming to perform PhD studies in this domain it will primarily be personal interest that defines a choice between the research lines identified above. However, further and even more detailed personal choices need to be made. First there is the choice between basic and fundamental versus more translational research; second, a choice for either technological or more disease mechanism-oriented studies needs to be made. Translational and technological research is what most companies in the infectious disease field will aim for and obviously targets can be at the biological, engineering and/or data-management levels. Depending on the selections depicted above, candidate PhD students may be more or less inclined to pursue either an academic or an industrial career.

Recent large-scale innovation in diagnostic clinical microbiology

- ELISA and other antigen–antibody tests developed from Western blot format to real point of care tests.
- PCR introduced from the eighties in the previous century onward, reaching diagnostic maturity in automated multiplexed, syndrome-based, nearly point of care testing formats.
- PCR developed into affordable, contamination-free high throughput assays.
- Introduction of mass spectrometry for bacterial identification generated a paradigm shift over the past decade; MS is considered the new gold standard for bacterial identification.
- Laboratory consolidation and ensuing laboratory automatization is becoming more customized.
- Acceptance of the relevance of microbiome analysis for infectious disease diagnostics.
- Enrichment of the antimicrobial susceptibility testing markets with a good number of small companies introducing new technologies.
- Introduction of next generation sequencing technology and innovative bio-informatics for tracing bacterial pathogens and their subtyping. Transformation of public health screening for tuberculosis towards NGS technology.
- Large scale use of patient-centric data for the prediction of improvement or deterioration of clinical situations.

## PhD studies in academia or industry?

A recent publication in Nature on career development for academics showed that for most graduated university students an academic career is quite unlikely to develop [6]. Only a 5% or lower minority of junior medical researchers will in the end succeed in academia, not based on the quality of the individual researchers but mostly because of lacking job opportunities. About 70% of the successful academics state to be very satisfied with their career choice. Interestingly, there are differing opinions on satisfaction at the salary level: only about 40% of PhD-possessing academic researchers are happy with their university salaries whereas in the industry this percentage is close to 60%. Problems faced by all academically or industrially employed researchers concern lack of funding, lack of permanent positions, gender disparities and other forms of discrimination, balancing professional and personal life, and dealing with anxiety or depression [7] (see Box 2 for a summary). For all of these different parameters there seem to be significant differences between men and women as well. to make an informed career choice, carefully considering a PhD student position in either academia or in industry is important. We here compare the pros and cons of accepting a PhD degree-providing research position in either industrial or academic settings.

In Defence of anacademic career in microbiology according to Schiloss [7] Despite

-  Low return on investment of grant applications.
-  Many e-mails and redundant meetings.
-  A persistent need for more funding.
-  Long-term training courses for use of simple equipment.
-  The need to publish in high impact journals.
-  Significant structural and social problems in university.

Still

-  Love to complain about the job.
-  Can study whatever I prefer to study and nobody will block that.
-  Have ownership of projects and can set all rules of the game.
-  Enjoy teaching.
-  Have 20% freedom to pursue non-campus work and projects.
-  Never had to punch a clock.
-  Feel to belong.

## “Free” academic choice?

Obtaining a PhD degree should preferably happen in a highly innovative, interdisciplinary and international high-quality research environment. However, the concept of the free academic choice is becoming less transparent: research, whether it is academic or industrial, requires funding and without funding even the brightest ideas may never be translated into any scientific and/or social practice. The disbalance between funding and financial requirements is a driving force towards targeted innovation rather than free-roaming exploration. Also the source of funding (university grant, grant by government other than academic, grant by Non-Governmental Organizations, grants provided by charities, etc.) is important with respect to where and how the research boundaries will be defined. Overall, there is an increasing tendency for research to be quite focused including pre-defined deliverables, where failure to deliver may have financial repercussions. It is good to have targets but this should not get in the way of creativity and the academic obligation to innovate and also explore ill-defined research avenues. At the end of the day, PhD students need to perform and in academia performance is mostly deduced from the perceived quality of the journals in which the student publishes. The better the journal, the more academically appreciated. High impact publications open doors into high-brow institutes for post-doc positions and help acquire prestigious grants. Still, in modern times it should no longer selectively be the creative publications that define success in the PhD process: communication skills, leadership, management of intellectual property, adaptive behavior, teaching quality, network development and internationalization continue to grow in importance.

## A recent european model for Phd studies

Over the coming years (2021–2027) the European Horizon research program will be investing over a hundred billion euro. The focus on Research and Development will change towards R&I, Research and Innovation. Projects need to deliver a better fit with regulatory aspects, market creation and early private investment. Infectious disease R&I will remain among the six focus areas.

The former Marie Curie fellowships, funded by the European Union, were individual grants that pushed mobility of individual researchers by demanding that grants acquired be spent in countries outside the native region of the PhD candidate. By having the PhD students cross national frontiers the scientific programs got the much required international dimension and besides the usual scientific issues the students faced logistic, cultural and travel and subsistence challenges as well. The previous Marie Curie scheme aimed at students developing an academic career mainly.

**Fig. 1:**
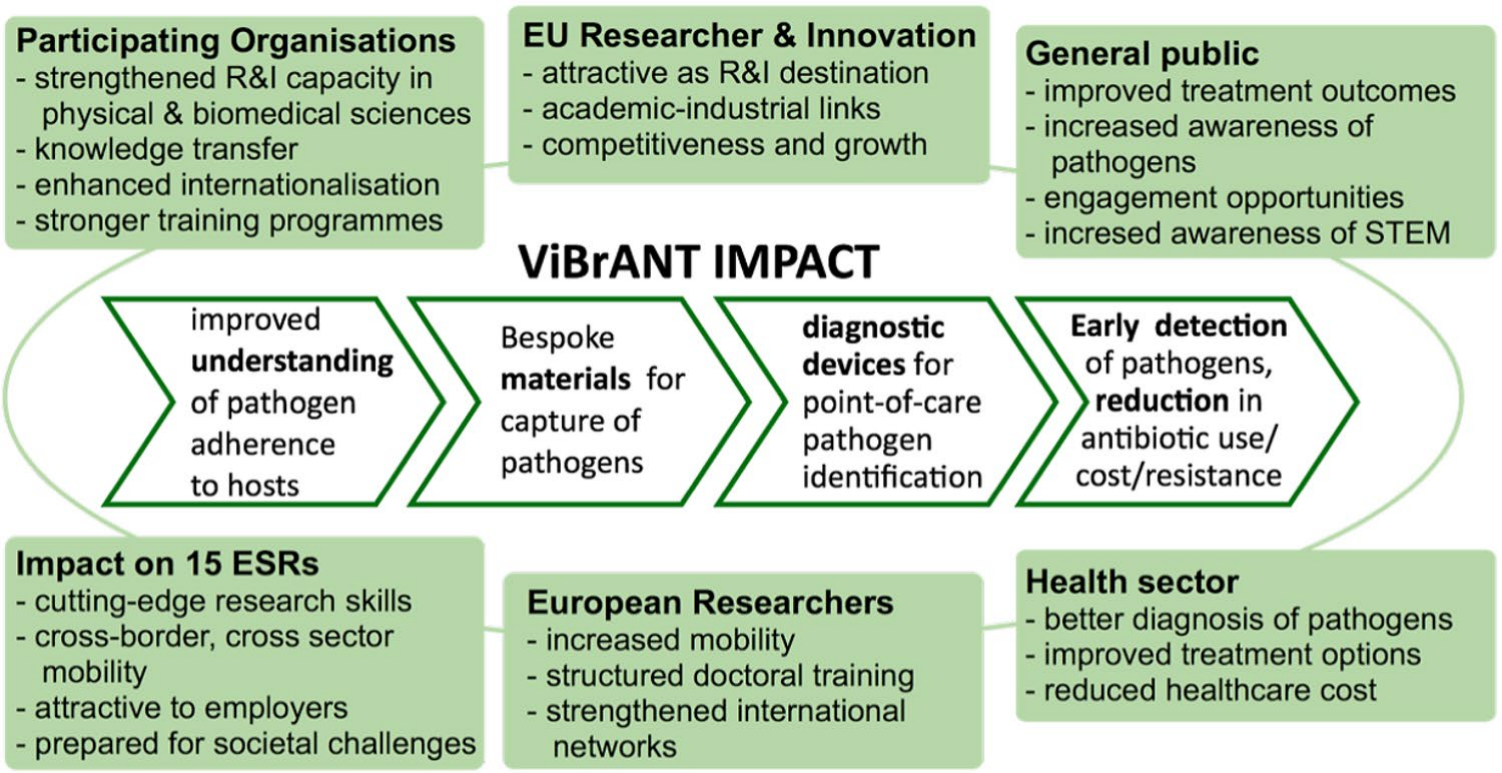
Socio-economic impact of ITN on viral and bacterial adhesion ViBrANT

The current Marie Curie granting scheme, remaining as one of the three open science pillars at the EU funding level, has yet another dimension: the grants are no longer individual and the grants finance a cohort of about 15 students, working on various aspects of a common theme. In addition, there is a choice of academic versus industrial employ. The international requirements have remained and every student has to work in a foreign country. In addition, the PhD program contains many specific features aimed at better visualization and dissemination of the work. For instance, dealing with the press and participating in mock or genuine press conferences may be part of the training program while dealing with modern means of mass-communication (Twitter, Blogs, Vlogs, maintaining a personal website, etc.) are obvious targets in the education of the next generation scientists. The personal granting scheme has been redeveloped into what are currently known as Innovative Training Networks (ITNs). These teams really have to function as such and at least bi-annual meetings are organized to exchange scientific and other experiences. Also, there is a requirement to spend part of the 3–4 year PhD program in yet another country. Laboratories of all partners in a consortium are open for secondments to be carried out by students employed by other teams in the group, providing excellent opportunities for exchange of expertise. Joint teaching events and the opportunity to organize a congress on consortium developments and findings are additional extras. The current Marie Curie networks promote collaborative innovation [8, 9]. Figure 1 exemplifies the structure and impact on PhD education of current ITNs and sketches the attractiveness of such programs for early-stage researchers.

Finally, the European R&I focus will significantly remain on Public–Private Partnerships (PPPs) as well. The initiative to try and move research closer to market will be pursued through PPPs which should also lead to the development of creative frameworks facilitating industrial-academic collaborations. Entrepreneurship will be promoted as a significant part of a PhD training. The Innovative Medicines Initiative (IMI) will provide another instrument for promoting public–private interactions with the biggest current budget available (more than 5.3 billion euro to be invested before 2025). IMI provides a tool for co-investment by the European Union and interested industries.

## Freedom to publish

Publication of research data is at the heart of every PhD project: students are judged by their scientific output and publishing in high-ranking journals is supposed to be a core activity. Open Access publication is a way to return science to society. Companies still have to deal with extreme precaution on what can be made public and what not. It seems though that the number of open-access publication in pharma has grown a lot [10] and the question is whether companies in the diagnostic domain should follow this lead. It has been advocated that publication for industry is a very efficient means for defining their roadmaps and strategies. Visibility at that level will attract new talent and will help acquire external funding. Scientific publication is also a good way to inform customers on new tests and recent developments and to support the quality perception of products.

External presentations and posters are other “physical” means to develop scientific visibility. Still, there are many alternative ways of validating research and in industrial settings there are two very important ones: the development of intellectual property (“patenting”) or the improvement or initial development of completely new products. In industrial settings the simple development of expertise or key internal or commercial tools and products can be essential to company survival or profitability. Normally, when patents have been deposited publications can be generated but in case of new products it may be needed to keep certain procedures or components really out of the public domain. This type of tactical approach may of course have an effect on PhD publication opportunities. Such industrial expertise often referred to as trade secrets, is extremely important, mostly very innovative and key to the survival of quite a few companies. The same holds true for product design, validation and verification: activities looked upon with dismay by many fundamental researchers but in themselves complicated, hard to manage and in need of high-level creativity and perseverance. This is why prior to release of data often marketing, legal and regulatory advice is needed to assure that patents are not harmed or infringed and that trade secrets are not unintendedly revealed. Doing a PhD study in such a context can be problematic depending on one’s personal interest. Performing the PhD exam in a confidential manner is an option, but takes away a big festive part of the celebration. Box 3 succinctly summarizes the various key steps in the development of innovative diagnostic tests. On a final note, it has to be emphasized that all levels of fundamental research are usually at the taxpayer’s credit and that basic science and commercial success are usually one a single step apart.

Key steps in the development of clinical diagnostic tests at the industrial level

- Define a project development plan for the diagnostic test.
  - Summarize the basic research fundamental to proper development.
  - Perform stakeholder mapping.
- Take a first business decision on the feasibility of the development plan.
  - Consider pricing environment and reimbursement barriers.
  - Competition monitoring and technology assessment.
  - Assemble a scientific advisory board and contact key opinion leaders.
- Develop a business proposal.
  - Draft a value dossier.
  - Design public affairs approaches.
  - Start market education activities.
  - Position the test in the current guidelines.
- Subject the proposal to a first business review.
  - Start health-economic studies.
- Perform feasibility studies and improve the design of the diagnostic test.
  - Set up collaborations with future users of the test to further validate.
- Fix the design of the test to be developed.
- Perform the second business review.
- Perform verification studies and start the go-to-market preparation.
- Perform validation studies with internal and external partners.
  - Perform clinical validation studies in the real-life environment.
- Design the commercialization activities.
- Product registration and launch.
  - Perform post marketing studies.

NB In black bullets the usual product development scheme is presented in a simplified, abbreviated manner.

## The individual choice

In the US the average number of scientific papers published per year declined significantly among industrial researchers over the past 35 years [11]. The same authors also claim that many large corporations withdrew from research and shifted their activities to less risky development processes, although in the life science sector this trend was less significant. This indicates better conditions in this sector for young researchers to pursue an industrial research career. Then again, career choices are hard to make and any support to facilitate individual choices should be much appreciated. There is obvious importance in the exploit of individual networks: discussing among one’s peers and future employers can be done in a variety of on-line formats (e.g. the professional LinkedIn network or the Indeed job opportunity site and the likes). In addition, there are targeted events where jobs are offered by employers and where future employees can meet and discuss with representatives of international companies and academic institutes, attend workshops, get educated on writing a CV and presenting oneself optimally for prospective employers and hence maximize career prospects for instance via the Nature Job Career Expo. An increasingly important category of employers are those in start-up or spin-off companies. These are usually more upfront in their research efforts and have a “young” culture where PhD students fit in well. In such cases, there needs to be a close collaboration or interaction with an academic institute since companies do not provide the PhD degrees. It is interesting to note that there are also clear advantages for academic institutes to generate and support start-up activities. MIT for instance generates about 5% of its annual research budget from revenues made by its associated start-up companies. One of the most positive points in working for a small company is that research data are mostly directly implemented for translational application [12].

## American academy of microbiology’s take on basic research

In a recent report by the AAM the academy states that graduate programs in the US are a single local source of trained scientists in the biological sciences and that difficulties exist in the communication of scientific knowledge between scientists and the public [13]. Graduate education will need to be improved and better aligned with the 21st century needs. Solutions are suggested to be in the outreach of biomedical researchers to “people in other disciplines” and professional societies are mentioned as an obvious source of much needed scientific skills. Apparently, there is a great need for better critical thinking, ethical consideration and experimental design. Finally one should better evaluate the impact of scientific discoveries and a steady “lifelong learning” attitude is a must. Maybe due to the limited number of industry participants in the AAM group and/or to differences between US and Europe in the involvement of industries in supporting PhD programs, in the AAM report the roles set apart for the companies are focused on setting up job-finding, job-interviewing and matchmaking events when in need of highly trained people. In fact, performing PhD research with an industrial partner offers many opportunities to learn and develop skills recommended by AAM (collaborative activities, risk assessment, project planning and development, general business etc.). In addition, with the rapidly increasing need for researchers trained in translational investigation, industrial involvement should develop even more over the years to come [12]. This also underscores the need for different models of evaluation of academic success: patents, track records in product development and even commercial success should be part of any research-oriented curriculum vitae.

## Conclusion

Choosing between academic or industrial opportunities for doing a PhD study is a complicated decision to make (see Table 1 for a brief analysis of advantages and drawbacks). Whatever the setting, a personal preference needs to be developed upon careful evaluation of all professional arguments, positive and negative ones. Assessment of one’s interest in fundamental research aspects versus more translational considerations when R&D or R&I is concerned are the most important driving factors. It is advisable that PhD students working with industry remain close to academic support, whereas for those PhD students working in academia, a solid business training should be part of the educational package on offer.

**Table 1:** Qualitative comparison of advantages and drawbacks of university-versus industry-based PhD programs

	University-based Phd positions	Industry-based Phd positions
PROs	All administrative conditions concerning a PhD project well managed making it easy to comply with local conditions and requirements Highly multi-disciplinary environment, collaborations on diverse subjects easy to trace Flexible management of content aspects and usually more time to finish a PhD program Specialised environment with extensive experience in the management of PhD students. Presence of colleague PhD students with whom to discuss functioning, problems and other challenges	Possibly better equipped laboratories Better compliance with regulatory conditions Good facilities for IP management. Better, sometimes more stable budgets Relatively fixed scientific programs, at least those closely affiliated to the development of the core product portfolio Mostly better wages and increased social coverage Acquiring optimal understanding of the business aspects of research
CONs	Small chances of local career development Budget restraints Non-pragmatic approaches and relative hobby-ism rather than focused research Internal competition for resources. A more difficult access to postdoc jobs in industry after the degree has been acquired	Need for an academic partnership to acquire an actual PhD degree: industries do not issue such degrees and there may be limited visibility on degree requirements An increasing number of tutors and supervisors (industrial and academic) which should have a positive scientific impact. Writing processes can become lengthy though Risk of project abrogation due to non-scientific reasons due to varying industrial priority setting based on e.g. customer or community demand Non-creative development projects may get in the way of research activities Difficulties in collecting the required Continuous Medical Education score
